# High BAL sRAGE is Associated with Low Serum Eosinophils and IgE in Children with Asthma

**DOI:** 10.3390/children7090110

**Published:** 2020-08-24

**Authors:** Jason T. Patregnani, Bonnie A. Brooks, Elizabeth Chorvinsky, Dinesh K. Pillai

**Affiliations:** 1 Division of Cardiac Critical Care Medicine, Children’s National Hospital, Washington, DC 20010, USA; 2 Department of Genomics and Precision Medicine, The George Washington University, Washington, DC 20052, USA; Ewilliams3@childrensnational.org (E.C.); Dpillai@childrensnational.org (D.K.P.); 3 Division of Critical Care Medicine, Children’s National Hospital, Washington, DC 20010, USA; Bbrooks3@childrensnational.org; 4 Division of Pulmonology, Children’s National Hospital, Washington, DC 20010, USA

**Keywords:** child, asthma, glycation end products, advanced, cytokines, immunoglobulin E, eosinophils

## Abstract

Asthma remains the most common chronic lung disease in childhood in the United States. The receptor for advanced glycation end products (RAGE) has been recognized as both a marker of and participant in pulmonary pathophysiology. While membrane-bound RAGE (mRAGE) perpetuates the type 2 immune response, the soluble form (sRAGE) may act as a decoy receptor for pro-inflammatory ligands. Bronchoalveolar samples from 45 pediatric patients with asthma were obtained. Patients were divided into high and low BAL sRAGE groups using median sRAGE. Descriptive statistical analysis and non-parametric testing were applied. Children in the “high” sRAGE group had a lower median serum eosinophil (0.27 [SE ± 0.04] vs. 0.57 [± 0.06] K/mcl, adjusted *p* = 0.003) and lower serum IgE level (194.4 [± 60.7] vs. 676.2 ± 140.5) IU/mL, adjusted *p* = 0.004) as compared to the “low” sRAGE group. When controlling for age and body mass index percentile, absolute eosinophil count (*p* = 0.03) and serum IgE (*p* = 0.043) remained significantly lower in the “high” sRAGE group. Children with asthma and high levels of BAL sRAGE have lower serum eosinophil and IgE levels. These findings are consistent with the hypothesis that sRAGE may act as a decoy receptor by binding ligands that normally interact with mRAGE.

## 1. Introduction

Asthma remains the most common chronic lung disease in childhood, affecting approximately six million children in the United States alone [1]. It is the third-ranking cause of hospitalization among children younger than 15 years and has an annual economic cost of over $81.9 billion dollars [2,3].

It is becoming increasingly apparent that asthma results from a complex interaction between a patients’ immune systems, the environment, and their underlying genetics [4]. Immunologically, the release of Th-2 pro-inflammatory cytokines leads to B-cell mediated production of IgE [4,5,6]. IgE is a known regulator of the chronic allergic response through the facilitation, processing, and uptake of antigens as well as modulating mast cell-mediated airflow obstruction [7,8]. Importantly, though IgE clearly plays a key role in the inflammation of chronic, allergic-type asthma, it has been realized that much of the inflammation itself is not IgE dependent. T-cells (specifically Th-2 type) and cytokines are also necessary for the eosinophilic infiltration of the small airways [9,10]. Full understanding of all the immunologic interactions that result in the asthmatic phenotype of airway inflammation, hypersecretion, and airway hyperresponsiveness remains elusive.

The receptor for advanced glycation end products (RAGE) is a multi-ligand transmembrane protein that is a member of the immunoglobulin super family and is highly expressed on type I alveolar cells in the lung [11,12,13]. It has been increasingly recognized as both a marker of and participant in multiple pulmonary pathophysiologic processes including acute respiratory distress syndrome and bronchiolitis [14,15,16]. RAGE works through multiple pathways including leukocyte adhesion, adaptive immunity, and inflammation through sustained activation of nuclear factor kappa B (NFkB) [17,18,19]. Upregulation of airway epithelial NFkB pathways has been shown to enhance airway hyperresponsiveness and mucus secretion in a mouse model of induced allergic inflammation. In addition, RAGE is critical to the accumulation of group 2 innate lymphoid cells to the lungs, an important process that highly contributes to the asthmatic phenotype. Taken together, it is highly suggestive that the RAGE axis is important in the underlying immune response for chronic, allergic-type asthma [20,21,22].

**Figure 1:**
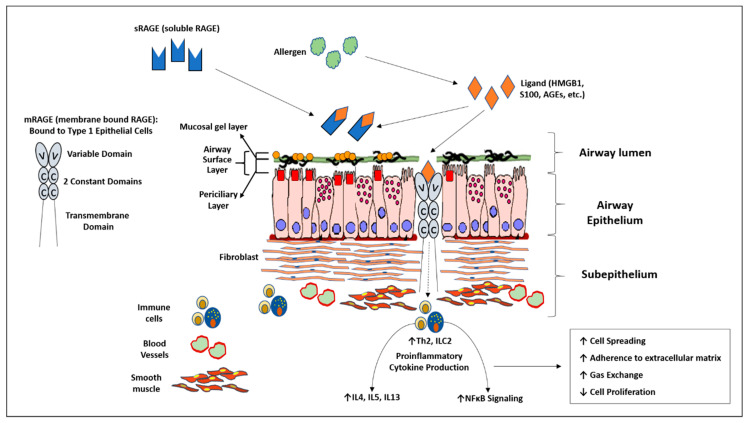
Diagram summarizing the receptor for advanced glycation end products (RAGE) inflammatory axis within the airway. Membrane bound RAGE (mRAGE) is a transmembrane receptor located predominately bound to type 1 epithelial cells of the lung. It binds multiple pro-inflammatory ligands resulting in downstream elevation of type Th2 cytokines and nuclear factor kappa B activity. Soluble RAGE contains similar ligand binding domains as mRAGE without the transmembrane/cytosolic portion of the receptor. It is thought to act as a decoy receptor by binding ligands destined for mRAGE.

RAGE exists in two major isoforms: membrane-bound (mRAGE) and soluble (sRAGE). mRAGE has been shown in animal models of asthma to perpetuate eosinophilia, a type 2 immune response in the lung, and is an early actor in sensitization to allergens [15,23,24]. sRAGE is thought to act as a decoy receptor for ligands destined for mRAGE [15,16,22]. This is due to the fact that sRAGE is known to retain the extracellular binding domain for these ligands after splicing [15,25,26]. sRAGE has been found in the sputum of both adult and pediatric asthmatics and can be found in the bronchoalveolar lavage fluid of patients with various pulmonary inflammatory processes [12,27,28,29]. When taken together, it appears that RAGE may be an important mediator in the pathophysiology of asthma. The RAGE pathway within the airway is summarized in Figure 1. We hypothesize that in children with moderate to severe asthma, high levels of bronchoalveolar lavage (BAL) sRAGE is associated with decreased serum eosinophilia and IgE.

## 2. Materials and Methods

Children undergoing bronchoscopy and evaluated in the Pulmonary Division’s Severe Asthma Program at Children’s National Hospital were enrolled in this study. All children in our program up to 21 years of age were included in the study. Moderate to severe asthma was defined using National Asthma Education and Prevention Program (NAEPP) guidelines for the diagnosis, classification, and management of asthma in children, which stratify asthma severity based on clinical impairment (daytime and nighttime symptoms, activity limitation, and lung function testing) [30]. Children with comorbid conditions that were excluded from this study include: chronic respiratory failure (*International Classification of Diseases*, Tenth Revision, code J96.10), ventilator dependence (Z99.11), tracheostomy dependence (Z93.0), chronic lung disease (J98.4), and cystic fibrosis (E84.xx). Similarly, patients with diagnoses related to cardiologic, immunologic, rheumatologic, diabetes, and neurologic disorders were excluded. This included congenital heart defects (*International Classification of Diseases*, Tenth Revision, code Q24.9), cardiomyopathy (I42.9), pericarditis (I31.9), myocarditis (I51.4), endocarditis (I33.0), valvular heart disease (I35.9), lupus (M32.9), rheumatoid arthritis (M06.9), Goodpasture’s (M31.0), granulomatosis with polyangiitis (M31.30), vasculitis (I77.6), primary immunodeficiency (D84.9), diabetes mellitus (E08.xx–E13.xx) and neuromuscular disease (G70.xx). All subjects and/or their family gave informed consent before they participated in the study. The study was conducted in accordance with the Declaration of Helsinki and the protocol was approved by the Institutional Review Board at Children’s National Hospital (Protocol number 01322). Flexible bronchoscopy with BAL was clinically indicated for refractory symptoms despite appropriate medical management. BAL samples were collected per our standard protocol. Briefly, after wedging a bronchoscope in 2nd generation bronchi or further, up to 2 mL/kg normal saline was instilled and recovered to obtain samples. BAL were spun at 500 g to remove cellular debris, and the supernatant was stored at −80 °C for future analysis. BAL samples were evaluated for percent of eosinophils, neutrophils, and presence of bacteria or viruses. Serum IgE and eosinophils, as well as BAL neutrophil and eosinophil cell count, were measured in Children’s National Hospital’s Division of Pathology and Laboratory Medicine accredited clinical laboratory. Total sRAGE was measured from the BAL samples using an enzyme-linked immunosorbent assay provided by MesoScale Discovery System (Rockville, MD, USA). The minimal detectable sRAGE level of the assay is 4pg/mL and samples were processed based on manufacturer’s instructions. Spirometry was measured in Children’s National Hospital’s accredited pulmonary diagnostics laboratory based on American Thoracic Society (ATS) criteria within 1 month of BAL collection [31]. No therapy was changed between the time of BAL and spirometry. Statistical analysis was performed using SPSS 25 (Chicago, IL, USA).

### Statistical Analysis

Children were divided into “high” and “low” BAL sRAGE groups using the median RAGE expression value (1760.7 [IQR 394.9, 3426]) pg/mL for the entire cohort. *T*-test and χ2 analysis were performed for normally distributed data, and non-parametric testing was performed when appropriate. Additionally, when performing correlation analysis for continuous variables with non-Gaussian distribution (BAL sRAGE, serum IgE, and serum eosinophils), data were log transformed prior to Pearson correlation evaluation. Power calculation for achieving 90% confidence in eliminating Type II error and a *p* value of <0.05 (Type I error) was based on expected eosinophils for “high” (0.25 K/mcL) and “low” (0.5 K/mcL) sRAGE, and a standard deviation of 0.25. Using these criteria, we estimated a minimum enrollment of 21 subjects in each group.

## 3. Results

Forty-five children were enrolled in the study. There were two important demographic differences between the “high” and “low” sRAGE groups: those in the “high” sRAGE group had a lower body mass index percentile 43.8 [IQR21.5-90.2] versus 87 [IQR43.6, 93.4]%ile; *p* = 0.042, Table 1 and a lower percentage of the comorbidity of eczema (21.7% vs. 50%, *p* = 0.048, Table 1). There was a similar trend toward reported allergic rhinitis, though this did not reach statistical significance (78.3% vs. 95.5%, *p* = 0.09, Table 1).

When comparing serum studies between those in the “high” versus “low” sRAGE groups, those children in the “high” sRAGE group had lower serum absolute eosinophils (0.27 ± 0.04 vs. 0.51 ± 0.06, *p* = 0.003, Table 2 K/mcL and lower IgE levels (194.4 ± 60.7 vs. 676.2 ± 140.5 *p* = 0.004, Table 2) IU/mL as compared to those in the “low” sRAGE group. BAL studies were performed on all patients with no significant difference in eosinophil counts or presence of viral or bacterial pathogens (Table 2). Spirometry was completed on 30 of 42 patients in the study and there were no significant differences seen between the “high” and “low” sRAGE groups Table 2.

**Figure 2:**
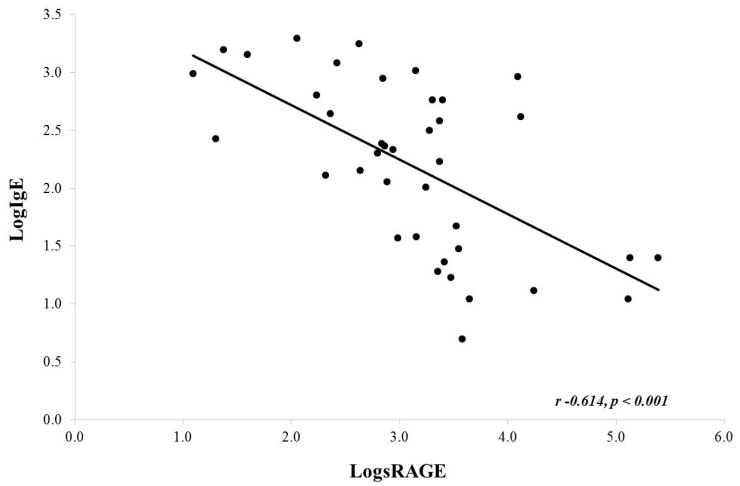
Correlation between serum IgE and BAL sRAGE. There was a significant negative correlation noted.

Upon correlation analysis using log-transformed data (due to non-Gaussian distribution), there was an inverse correlation noted between serum IgE and BAL sRAGE (Pearson, *r* = −0.614, *p* < 0.001) (Figure 2). A similar analysis for serum absolute eosinophils and BAL sRAGE was not significant (Pearson, *r* = −0.214, *p* = 0.192). It should be noted that serum eosinophils were close to normally distributed. If we do not log transform eosinophils, we do find a similar significant inverse correlation (Pearson *r* −0.430, *p* value 0.006; data not shown).

**Figure 3:**
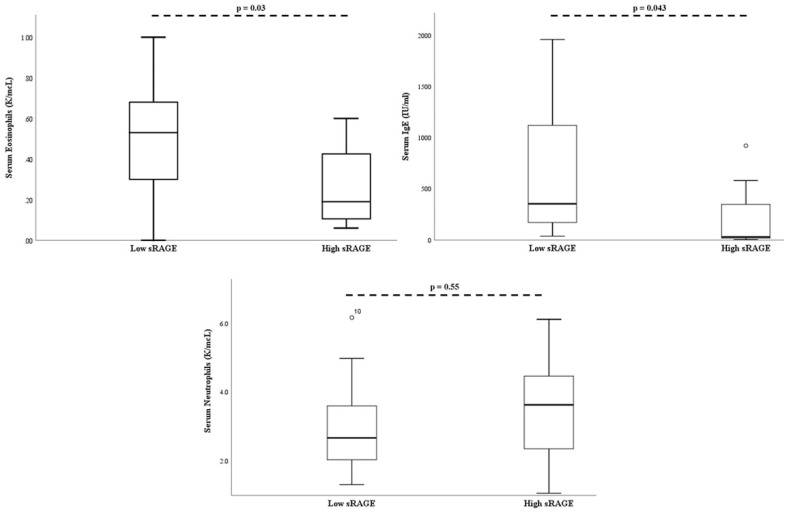
Bronchial alveolar lavage soluble RAGE is associated with decreased serum eosinophils and serum IgE. There was no association between soluble RAGE and serum neutrophil levels. *P*-values adjusted for age and BMI percentile. The open-circle denotes subjects outside the 95% confidence interval.

On multivariate analysis, when controlling for age and body mass index percentile, serum absolute eosinophil count (*p* = 0.03), and serum IgE (*p* = 0.043) remained significantly lower in the “high” sRAGE group (Figure 3).

## 4. Discussion

This is the first study evaluating sRAGE in the BAL fluid of children with moderate to severe asthma. We found that those children with higher levels of BAL sRAGE had decreased serum eosinophil and IgE levels. We did not identify any difference in serum neutrophil levels. These finds are consistent with the proposed mechanism that sRAGE can act as a scavenger or decoy receptor for ligands that are destined for mRAGE on the alveolar cell surface. Currently, it is thought the inflammatory cascade mediated through RAGE begins with allergens inducing ligands that are binding to mRAGE including HMGB1 and S100A. Both of these ligands have been shown to recruit eosinophils to the lungs and higher levels in sputum have been correlated to increased severity of the inflammatory response in the lungs [32,33]. In mouse models of allergic asthma, RAGE knockout (KO) mice were protected against the phenotype of airway eosinophilia, goblet cell hyperplasia, and decreased pulmonary function [22]. In addition, RAGE KO mice were noted to be deficient in IL-5 and IL-13 while wild-type animals readily produced these type-2 cytokines in response to allergens, suggesting a key role for RAGE in the Th2 inflammatory cascade [22]. In the same study, sRAGE was also administered as a therapeutic agent and it was noted that the asthmatic phenotype was rescued. Ultimately, this suggests that sRAGE may act as a decoy receptor as hypothesized [22].

In humans, studies examining the role of sRAGE in the pathogenesis of asthma are limited. In a study of moderate to severe asthmatic adults, BAL fluid in moderate to severe asthmatics showed negligible levels of sRAGE, though importantly, this study examined those asthmatics with a neutrophilic phenotype [34]. This is in contrast to the study by Watanabe et al., which found higher levels of a subtype of sRAGE in the sputum of asthmatics with a neutrophilic predominance [27]. In our study, we did not identify any association between sRAGE and the presence of viral (n = 12; *p* = 0.26) or bacterial (n = 6; *p* = 0.64) pathogens in BAL. We also did not note any difference in neutrophils between the two groups in our study (*p* = 0.54). In children specifically, serum sRAGE levels have been shown to be lower in patients with asthma compared to control patients. In the same study, there was a significant positive correlation between sRAGE levels and FEV1, as well as a negative correlation between serum sRAGE, eosinophil count, and total IgE [28]. As our study did not include serum levels of sRAGE and only BAL fluid levels, we do not know if BAL and serum levels correlate in our population. Kamo et al. found a similar lack of relationship between BAL fluid and serum sRAGE in adult patients with ARDS and/or other respiratory infections. They speculate that this may be due to the capillary leak generally noted as part of these disease processes [35], but further study into how sRAGE is spliced and present in different “compartments” requires further study.

Our study also showed that children in the “high sRAGE” group also had a lower rate of other atopic diseases such as eczema (22% vs. 50%). Though no study has examined if RAGE specifically participates in pathophysiology of eczema in children, HMGB1, a main ligand in the RAGE inflammatory axis, has been shown to be significantly higher in children with eczema and its level is related to the severity of clinical disease [36]. We also found a trend toward significance for a lower rate of allergic rhinitis in the “high sRAGE” group (78% vs. 96%, *p* = 0.09) which can potentially be explained in a similar way. The one study that examined the possible role of RAGE in allergic rhinitis found high levels of mRNA expression of HMGB1 in nasal brushings of those with allergic rhinitis compared to those that did not, though levels of RAGE were not different between the two groups [37]. As the RAGE inflammatory axis plays in important role in the initiation and perpetuation of allergic-type asthma, it is probable that it has a similar role in these other atopic diseases that have similar pathophysiologic mechanisms.

In addition to the relationships between sRAGE, asthma, and atopy noted above, RAGE is becoming increasingly studied in a wide range of disease processes from metabolic syndrome, to coronary artery disease, as well as multiple forms of cancers [38,39]. We found that those children in the “high” RAGE group had a lower BMI percentile as compared to those in the “low” RAGE group. It has been shown in adults that those with lower median serum sRAGE have worse signs of metabolic syndrome, specifically around elevated waist circumference, higher blood pressure, and higher fasting glucose levels [39]. The significance of higher BAL sRAGE on BMI in this population is unclear, though if BAL levels correlate with serum levels, this would be consistent with adult data.

There are several limitations to our study, the largest of which is that we do not have serum levels of sRAGE or Th2 cytokines to correlate with BAL levels. Furthermore, we do not have BAL Th2 cytokines, nor serum or BAL Th1 cytokines to compare the impact of our sRAGE findings in a more global perspective. This will be quite important for future studies so that we may better understand the role of sRAGE and whether this decoy receptor is acting in the lungs or on a systemic level. In addition, our sample size is small; though for a pediatric study including BAL samples, the power appears adequate to show our hypothesized findings. Lastly, our study population is comprised of children diagnosed with moderate to severe asthma only. As this is the primary group that receives bronchoscopy, this study could not be done in more mild asthmatics or be compared to non-asthmatic children. Future studies could include this population by measuring sputum and/or serum sRAGE to understand the correlation between sRAGE in BAL fluid as compared to that in serum.

## 5. Conclusions

Children with moderate/severe asthma and high levels of BAL sRAGE show lower levels of serum eosinophils and IgE levels. This is consistent with the hypothesis that sRAGE may be acting as a decoy receptor for inflammatory ligands that are destined for mRAGE. As there may be a potential therapeutic implication for sRAGE in decreasing the inflammation in children with asthma and other atopic diseases, further investigation of the RAGE inflammatory axis is warranted.

## Figures and Tables

**Table 1:** Demographics.

Characteristic	Overall (n = 45)	Low RAGE (n = 22)	High RAGE (n = 23)	*p*-Value
Median age, year (IQR)	8.0 (4.0, 10.3)	8.5 (5.8, 11.1)	7.2 (2.6, 9.8)	0.41
Gender, % female (n)	26.7 (12)	31.8 (7)	21.7 (5)	0.45
Ethnicity				0.25
African America	73.3 (33)	81.8 (18)	65.2(15)	
Caucasian	15.6 (7)	9.1 (2)	21.7 (5)	
Other	11.1 (5)	9.1 (2)	13 (3)	
Gestational age, median week (n = 44)	40 (34.8, 40)	40 (34, 40)	40 (36, 40)	0.17
BMI percentile, median (IQR)	78.7 (25.2, 93.4)	87 (43.6, 93.4)	43.8 (21.5, 90.2)	**0.042 ^a^**
Reported diagnosis, % (n)				
Rhinitis	82.4 (42)	95.5 (21)	78.3 (18)	0.09
Eczema	35.3 (18)	50 (11)	21.7 (5)	**0.048 ^a^**
Reflux	35.3 (18)	31.8 (7)	39.1 (9)	0.61
Sleep-disordered breathing	23.5 (12)	18.2 (4)	30.4 (7)	0.34
Severe asthma	55.6 (25)	100 (22)	43.5 (10)	0.095
Median daily inhaled steroid dose, (fluticasone equivalent), mcg (IQR)	550 (500, 1050)	1050 (550, 1050)	550 (500, 1050)	0.102

^a^ = *p* < 0.05 considered statistical significance. Continuous variables calculated based on t-test. Categorical data calculated using χ^2^ testing. Ethnicity calculated using ordinal regression analysis. *p* Values for characteristics expressed in median values analyzed using Mann–Whitney U-test. BMI = body mass index, IQR = interquartile range, RAGE = receptor for advanced glycation end products.

**Table 2:** Patient outcomes.

Spirometry	Overall (n = 30)	Low RAGE (n = 18)	High RAGE (n = 12)	*p*-Value	
Median FEV1% predicted (SE)	87.9 (3.1)	86.9 (4.7)	89.4 (3.3)	0.7	
Median FEV1/FVC (SE)	84.2 (2.6)	83.7 (4.1)	84.9 (2.6)	0.83	
Median FEF25-75% predicted (SE)	70.7 (4.9)	66.9 (6.9)	76.4 (6.5)	0.35	
**BAL Studies**	**Overall (n = 45)**	**Low RAGE (n = 22)**	**High RAGE (n = 23)**	***p*-Value**	
Eosinophil %, mean (SE), n = 38	6.0 (1.4)	5.3 (1.4)	6.7 (2.4)	0.63	
Neutrophil %, mean (SE), n = 37	28.1 (4.2)	25.4 (7.0)	30.7 (5.0)	0.54	
+Viral Panel ^c^, % (n); n = 44		28.6 (6)	26.1 (6)	0.85	
+Bacterial culture % (n); n = 37		15.8 (3)	16.7 (3)	0.94	
**Serum Studies**					**Adjusted *p*-Value ^b^**
Serum IgE (IU/mL), mean (SE), n = 39	441.5 (86.3)	676.2 (140.5)	194.4 (60.7)	**0.004 ^a^**	**0.043 ^a^**
CBC absolute eosinophil (K/mcL), mean (SE), n = 40	0.39 (0.04)	0.51 (0.06)	0.27 (0.04)	**0.003 ^a^**	**0.03 ^a^**
CBC absolute neutrophils (K/mcL), mean (SE), n = 39	3.20 (0.22)	2.95 (0.28)	3.28 (0.34)	0.24	0.55

^a^ = statistically significant. ^b^ = adjusted for age and BMI percentile. ^c^ = *p* Value analyzed using χ^2^. *p* values for outcomes expressed as mean values analyzed using *t*-test. BMI = body mass index, BAL = bronchial alveolar lavage, CBC = complete blood count, FEV1 = Forced expiratory volume in first second, FVC = forced vital capacity, FEF = forced expiratory flow, IU = international units, mcl = microliter, mL = milliliter RAGE = receptor for advanced glycation end products, SE = standard error.
